# *N*-1 regioselective Michael-type addition of 5-substituted uracils to (2-hydroxyethyl) acrylate

**DOI:** 10.1186/1860-5397-3-40

**Published:** 2007-11-08

**Authors:** Sławomir Boncel, Dominika Osyda, Krzysztof Z Walczak

**Affiliations:** 1 Department of Organic Chemistry, Biochemistry and Biotechnology Silesian University of Technology, Krzywoustego 4, PL-44100 Gliwice, Poland

## Abstract

*N*-1 regioselective Michael-type addition of 5-substituted uracils to (2-hydroxyethyl) acrylate is presented. The reactions were performed in polar aprotic solvents and with avoidance of polymerization of acrylic substrate. The obtained adducts may serve as versatile substrates for further functionalization, e.g. into (3-uracil-1-yl)propanoic acids or transformations, with participation of hydroxyl group, into ester-conjugated acyclic nucleosides.

## Background

Nowadays, intensive research on the preparation of novel synthetic nucleosides plays an important role in the research scopes of academic bioorganic centres. Special attention has been reserved in the current synthesis literature for acyclic nucleosides.[1] Acyclic units containing hydroxyl groups are capable of phosphorylation and building into a growing nucleic acid, thus perturbing its replication. Several acyclic nucleosides, like HEPT (1-[2-hydroxyethoxymethyl]-6-(phenylthio)thymine),[2] or MKC-442 (1-ethoxymethyl-5-isopropyl-6-benzyluracyl) are in the third stage of clinical testing for their inhibition of HIV-1 reverse transcriptase.[3] Others, like acyclovir or gancyclovir are in clinical use.[4] Cidofovir, (*S*)-3-hydroxy-2-phosphonomethoxypropyl cytosine, is an acyclonucleoside that possesses broad-spectrum activity against numerous DNA-viruses.[5]

One of the most convenient methods of uracil ring *N*-alkylation is the Michael-type addition. Other general ways of alkylation are: nucleophilic substitution of halogenoalkyl substrates by activated uracil rings (in the *N*-anionic or *O*-persililated derivatives);[6–7] Mitsunobu reaction;[8] or reactions that operate through ANRORC-mechanisms that require the presence of strongly electron-withdrawing groups.[9] Recently, we have described an *N*1- and *N*3-regioselective Michael-type addition of 5-substituted uracil derivatives to methyl acrylate and acrylonitrile.[10] Following our aims, to synthesise ester-conjugated nucleosides, we would like to present an expedient method of uracil ring alkylation, with the potential for further functionalization towards acyclic nucleosides.

2-Hydroxyethyl acrylate (HEA), being a widely exploited monomer in the production of hydrophilic polymer gels,[11] has not been considered frequently as a potential Michael acceptor in organic synthesis.[12–13] We have developed an effortless, efficient and useful pathway for the synthesis of 2-hydroxyethyl-3-(uracil-1-yl)propanoates. The products of addition may be further functionalized e.g. at the 3-position of the uracil ring, at the hydroxyl group of the acyclic moiety or hydrolysed to 3-(uracil-1-yl)propionic acids.

## Results and Discussion

**Scheme 1:**
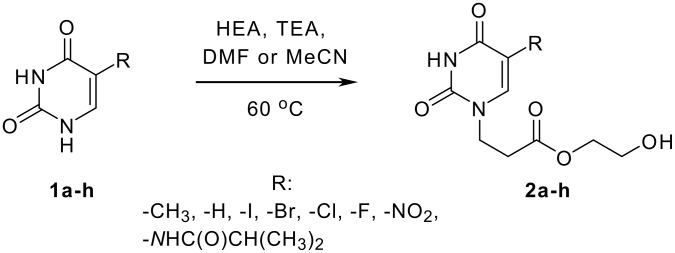
Michael-type addition of 5-substituted uracil derivatives to 2-hydroxyethyl acrylate.

Michael donors, namely uracil, thymine, 5-halogenouracils (5-fluoro-, 5-chloro-, 5-bromo-, 5-iodouracil), and 5-nitrouracil are commercially available, and 5-isobutyrylaminouracil was synthesized as reported previously.[14] Commercially available HEA was applied as the Michael acceptor (Scheme 1). Triethylamine (TEA) (1 equivalent) served as a deprotonating agent.

The addition reactions were performed in polar aprotic solvents such as DMF or MeCN (Table 1, footnote). The avoidance of polymerization of acrylic substrates was achieved by portion-wise addition to the reaction mixture. Moreover, addition of polymerization inhibitors, explicitly hydroquinone, did not cause any significant changes in polymerization rate. The optimum reaction temperature was found to be 60°C. The reactions were carried out until complete conversion of uracil substrate was achieved, as confirmed by TLC. Michael adducts were obtained in good to excellent yields (Table 1). See Supporting Information File 1 for full experimental data.

**Table 1:** Conditions for the Michael-type addition of 5-substituted uracil derivatives to 2-hydroxyethyl acrylate

No.	R	*pK_a_*[15]	A/D ratio*	*t* [h]	Yield [%]

1	-CH_3_ (**2a**)	9.77	1.2	7.5	98^[a]^
2	-H (**2b**)	9.44	2.0	20	41^[b]^
3	-I (**2c**)	8.13	1.2	7.5	59^[b]^
4	-Br (**2d**)	8.06	1.2	15	73^[b]^
5	-Cl (**2e**)	8.02	1.2	7.5	91^[b]^
6	-F (**2f**)	7.95	1.2	7.5	98^[b]^
7	-NO_2_ (**2g**)	5.50	3.0	39	73^[a]^
8	NHC(O)CH(CH_3_)_2_ (**2h**)	-	2.0	11.5	93^[a]^

^[a]^ Reactions performed in DMF ^[b]^ Reactions performed in MeCN *Acceptor/Donor ratio

Only in the cases of unsubstituted uracil (Table 1, Entry 2) and 5-nitrouracil (Table 1, Entry 7) did the reaction times have to be extended significantly. This was caused, in the first case, by the poor solubility of uracil in MeCN and, in the second one, by low nucleophilicity (correlated with the lowest *pK_a_* value among all uracils) towards conjugated acrylic system. In the other experiments, no correlation of acidity with reaction rate was observed.

**Scheme 2:**
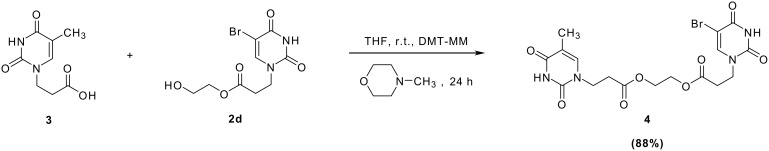
Synthesis of the model ester-conjugated acyclic nucleoside.

The obtained Michael adducts may serve as advantageous compounds for further functionalization or as substrates in the synthesis of ester-conjugated nucleosides. As an example of the transformation of an ester group, acidic hydrolysis of representative model compound **2a** to 3-(3,4-dihydro-5-methyl-2,4-dioxopyrimidin-1(2*H*)-yl)propanoic acid (**3**) (conditions of hydrolysis – see Supporting Information File 1) is additionally reported. In turn, as the representative way for the utilization of synthetic subunits, we have obtained ester-conjugated acyclic nucleoside, namely 3-(5-methyl-2,4-dioxo-3,4-dihydro-2*H*-pyrimidin-1-yl)-propionic acid 2-[3-(5-bromo-2,4-dioxo-3,4-dihydro-2*H*-pyrimidin-1-yl)-propionyloxy]-ethyl ester (**4**) (Scheme 2).

The reaction was performed in THF at room temperature, in the presence of 4-(4,6-dimethoxy-1,3,5-triazin-2-yl)-4-methylmorpholinium chloride (DMT-MM) which is a frequently used condensing agent.[16] The condensation required 1.1 equivalent of *N*-methylmorpholine as a co-catalyst. See Supporting Information File 1 for the detailed procedure. Structure of the product was confirmed by ^1^H and ^13^C NMR spectroscopy. See Figure 1 in Supporting Information File 1 for the assignment of particular protons in NMR spectrum of the model ester-conjugated nucleoside.

**Figure 1:**
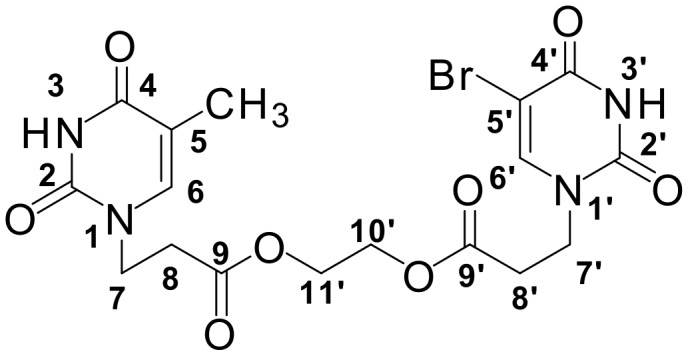
Protons assignment in NMR spectrum of the model ester-conjugated nucleoside (**4**).

## Conclusion

In summary, a simple and efficient method of *N*-1 alkylation of uracil rings using Michael-type addition has been elaborated. The complete regioselectivity has been attained in the presence of TEA as base. The tendency for polymerization of HEA has been successfully prevented. The application of the obtained adducts in further synthesis has been demonstrated.

## Supporting Information

File 1 Experimental. The data provides procedures, physical properties and 1H and 13C NMR spectra of all newly synthesized compounds.
