# Supplementary material for: N-1 regioselective Michael-type addition of 5-substituted uracils to (2-hydroxyethyl) acrylate
Source: Beilstein J Org Chem. 2007 Nov 8;3:40. doi: 10.1186/1860-5397-3-40 (PMC2151075; doi:10.1186/1860-5397-3-40)
Supplement: File 1 — Experimental. The data provides procedures, physical properties and 1H and 13C NMR spectra of all newly synthesized compounds. [file Beilstein_J_Org_Chem-03-40-s001.doc]

Experimental

**General:** NMR spectra were recorded at 300 MHz for ^1^H NMR and 75.5 MHz for ^13^C NMR on a Varian Inova 300 MHz in [D_6_]DMSO solution; *δ* values are in parts per million relative to tetramethylsilane as an internal standard. Elemental analyses were obtained using a Perkin-Elmer 240C apparatus. All used reagents were purchased from Aldrich. TLC 60F_254_ plates and silica gel 60 (0.040-0.063 mm) were purchased from Merck. Melting points were measured at Boetius apparatus and are uncorrected. DMF and MeCN were distilled prior to use and stored over molecular sieves 4A.

**General procedure:** To a round-bottomed flask immersed in an oil-bath and placed on a magnetic stirrer, 5-substituted uracil (1.0 mmol) and 10 ml of solvent (Table 1) were added. To this mixture triethylamine (1.0 mmol) and, after next 5 minutes, (2-hydroxyethyl) acrylate (0.6 mmol) were added dropwise while stirring. The mixture was heated and kept at temperature of 60 ºC during 2.5 hours. Afterwards, next portion of (2-hydroxyethyl) acrylate (0.6 mmol) was added and the heating was continued for the next 5-6 hours. When TLC revealed presence of limiting substrate, next portion of Michael acceptor was added until no longer presence of uracil was confirmed (Table). Subsequently, the solvent was evaporated under reduced pressure. Crude crystals were macerated with ethyl acetate, filtered off, dried on air and recrystallized from mixture of ethanol and ethyl acetate. In the case of **2g** and **2h**, products were isolated using column chromatography with 10% MeOH/CHCl_3_ as the eluting system.

**2-Hydroxyethyl 3-(5-methyl-2,4-dioxo-3,4-dihydropirimidin-1(2*H*)-yl)propanoate (2a):**

White crystals, m.p. 127-129 °C. ^1^H NMR (300 MHz, [D_6_]DMSO): *δ* = 11.26 (s, 1 H, N3-H), 7.51 (s, 1 H, 6-H), 4.83 (t, ^3^*J*_H,H_ = 5.5 Hz, 1 H, OH), 4.03 (t, ^3^*J*_H,H_ = 4.9 Hz, 2 H, OCH_2_), 3.85 (t, ^3^*J*_H,H_ = 6.7 Hz, 2 H, NCH_2_), 3.55 (q, ^3^*J*_H,H_ = 5.1 Hz, 2 H, *CH_2_*OH), 2.69 (t, ^3^*J*_H,H_ = 6.7 Hz, 2 H, CH_2_CO), 1.73 (s, 3 H, CH_3_) ppm. ^13^C NMR (75 MHz, [D_6_]DMSO): *δ* = 170.82, 164.30, 150.70, 141.83, 108.20, 66.01, 58.83, 43.82, 32.78, 11.93 ppm. C_10_H_14_N_2_O_5_ (242.23): calcd. C 49.59, H 5.83, N 11.56; found C 49.35, H 5.63, N 11.17.

**2-Hydroxyethyl 3-(2,4-dioxo-3,4-dihydropirimidin-1(2*H*)-yl)propanoate (2b)**:

White crystals, m.p. 95-97 °C. ^1^H NMR (300 MHz, [D_6_]DMSO): *δ* = 11.26 (s, 1 H, N3-H), 7.63 (d, ^3^*J*_H,H_ = 8.1 Hz, 1 H, 6-H), 5.53 (d, ^3^*J*_H,H_ = 7.7 Hz, 1 H, 5-H), 4.82 (t, ^3^*J*_H,H_ = 5.4 Hz, 1 H, OH), 4.04 (t, ^3^*J*_H,H_ = 5.2 Hz, 2 H, OCH_2_), 3.89 (t, ^3^*J*_H,H_ = 6.6 Hz, 2 H, NCH_2_), 3.56 (q, ^3^*J*_H,H_ = 5.2 Hz, 2 H, *CH_2_*OH), 2.71 (t, ^3^*J*_H,H_ = 6.8 Hz, 2 H, CH_2_CO) ppm. ^13^C NMR (75 MHz, [D_6_]DMSO): *δ* = 170.89, 163.80, 150.90, 146.15, 100.70, 66.06, 58.89, 44.13, 32.71 ppm. C_9_H_12_N_2_O_5_ (228.20): calcd. C 47.37, H 5.30, N 12.28; found C 47.55, H 5.15, N 12.01.

**2-Hydroxyethyl 3-(5-iodo-2,4-dioxo-3,4-dihydropirimidin-1(2*H*)-yl)propanoate (2c):**

White crystals, m.p. 115-117 °C. ^1^H NMR (300 MHz, [D_6_]DMSO): *δ* = 11.65 (s, 1 H, N3-H), 8.17 (s, 1 H, 6-H), 4.82 (t, ^3^*J*_H,H_ = 5.5 Hz, 1 H, OH), 4.04 (t, ^3^*J*_H,H_ = 5.1 Hz, 2 H, OCH_2_), 3.90 (t, ^3^*J*_H,H_ = 6.7 Hz, 2 H, NCH_2_), 3.56 (q, ^3^*J*_H,H_ = 5.2 Hz, 2 H, *CH_2_*OH), 2.72 (t, ^3^*J*_H,H_ = 6.6 Hz, 2 H, CH_2_CO) ppm. ^13^C NMR (75 MHz, [D_6_]DMSO): *δ* = 170.82, 161.09, 150.64, 150.42, 67.73, 66.02, 58.85, 44.17, 32.54 ppm. C_9_H_11_IN_2_O_5_ (354.10): calcd. C 30.53, H 3.13, N 7.91; found C 30.29, H 3.02, N 7.62.

**2-Hydroxyethyl 3-(5-bromo-2,4-dioxo-3,4-dihydropirimidin-1(2*H*)-yl)propanoate (2d):**

White crystals, m.p. 133-135 °C. ^1^H NMR (300 MHz, [D_6_]DMSO): *δ* = 11.78 (s, 1 H, N3-H), 8.18 (s, 1 H, 6-H), 4.82 (t, ^3^*J*_H,H_ = 5.5 Hz, 1 H, OH), 4.04 (t, ^3^*J*_H,H_ = 4.9 Hz, 2 H, OCH_2_), 3.92 (t, ^3^*J*_H,H_ = 6.7 Hz, 2 H, NCH_2_), 3.56 (q, ^3^*J*_H,H_ = 5.1 Hz, 2 H, *CH_2_*OH), 2.73 (t, ^3^*J*_H,H_ = 6.7 Hz, 2 H, CH_2_CO) ppm. ^13^C NMR (75 MHz, [D_6_]DMSO): *δ* = 170.84, 159.71, 150.27, 145.86, 94.31, 66.06, 58.87, 44.36, 32.46 ppm. C_9_H_11_BrN_2_O_5_ (307.10): calcd. C 35.20, H 3.61, N 9.12; found C 35.55, H 3.23, N 8.99.

**2-Hydroxyethyl 3-(5-chloro-2,4-dioxo-3,4-dihydropirimidin-1(2*H*)-yl)propanoate (2e):**

White crystals, m.p. 146-147 °C. ^1^H NMR (300 MHz, [D_6_]DMSO): *δ* = 11.80 (br. s, 1 H, N3-H), 8.12 (s, 1 H, 6-H), 4.82 (br. s, 1 H, OH), 4.04 (t, ^3^*J*_H,H_ = 5.0 Hz, 2 H, OCH_2_), 3.91 (t, ^3^*J*_H,H_ = 6.9 Hz, 2 H, NCH_2_), 3.56 (br. s, 2 H, *CH_2_*OH), 2.73 (t, ^3^*J*_H,H_ = 6.8 Hz, 2 H, CH_2_CO) ppm. ^13^C NMR (75 MHz, [D_6_]DMSO): *δ* = 170.80, 159.53, 150.03, 143.48, 105.83, 66.04, 58.84, 44.36, 32.42 ppm. C_9_H_11_ClN_2_O_5_ (262.65): calcd. C 41.16, H 4.22, N 10.67; found C 41.44, H 4.09, N 10.47.

**2-Hydroxyethyl 3-(5-fluoro-2,4-dioxo-3,4-dihydropirimidin-1(2*H*)-yl)propanoate (2f):**

Light grey crystals, m.p. 124-126 °C. ^1^H NMR (300 MHz, [D_6_]DMSO): *δ* = 11.76 (br. s, 1 H, N3-H), 8.05 (d, ^3^*J*_F,H_ = 7.0 Hz, 1 H, 6-H), 4.83 (br. s, 1 H, OH), 4.04 (t, ^3^*J*_H,H_ = 5.0 Hz, 2 H, OCH_2_), 3.86 (t, ^3^*J*_H,H_ = 6.9 Hz, 2 H, NCH_2_), 3.56 (q, ^3^*J*_H,H_ = 5.0 Hz, 2 H, *CH_2_*OH), 2.72 (t, ^3^*J*_H,H_ = 6.8 Hz, 2 H, CH_2_CO) ppm. ^13^C NMR (75 MHz, [D_6_]DMSO): *δ* = 170.77, 157.47 (d, ^2^*J*_F,C_ = 25.7 Hz, 1 C, 4-C), 149.50, 139.30 (d, ^1^*J*_F,C_ = 228.6 Hz, 1 C, 5-C), 130.60 (d, ^2^*J*_F,C_ = 33.8 Hz, 1 C, 6-C), 66.05, 58.85, 44.16, 32.44 ppm. C_9_H_11_FN_2_O_5_ (246.19): calcd. C 43.91, H 4.50, N 12.38; found C 44.09, H 4.42, N 12.21.

**2-Hydroxyethyl 3-(5-nitro-2,4-dioxo-3,4-dihydropirimidin-1(2*H*)-yl)propanoate (2g):**

White crystals, m.p. 113-114 °C. ^1^H NMR (300 MHz, [D_6_]DMSO): *δ* = 12.08 (br. s, 1 H, N3-H), 9.25 (s, 1 H, 6-H), 4.81 (br. s, 1 H, OH), 4.13-4.06 (m, ^3^*J*_H,H_ = 7.0 Hz, ^3^*J*_H,H_ = 5.0 Hz, 4 H, NCH_2_, OC*H*_2_CH_2_), 3.56 (t, ^3^*J*_H,H_ = 5.1 Hz, 2 H, *CH_2_*OH), 2.80 (t, ^3^*J*_H,H_ = 6.7 Hz, 2 H, C*H*_2_CO) ppm.

^13^C NMR (75 MHz, [D_6_]DMSO): *δ* = 170.78, 155.06, 151.36, 149.31, 124.62, 66.14, 58.85, 45.45, 32.14 ppm. C_9_H_11_N_3_O_7_ (273.20): calcd. C 39.57, H 4.06, N 15.38; found C 39.84, H 4.17, N 15.06.

**2-Hydroxyethyl 3-[5-Amino-5-(2-methyl-propion-1-yl)-2,4-Dioxo-3,4-dihydropirimidin-1(2*H*)-yl)propanoate (2h):**

White crystals, m.p. 116-117 °C. ^1^H NMR (300 MHz, [D_6_]DMSO): *δ* = 11.64 (s, 1 H, N3-H), 8.99 (s, 1 H, C5-N*H*), 8.35 (s, 1 H, 6-H), 4.78 (t, ^3^*J*_H,H_ = 5.7 Hz, 1 H, OH), 4.01 (t, ^3^*J*_H,H_ = 5.0 Hz, 2 H, OCH_2_), 3.90 (t, ^3^*J*_H,H_ = 6.6 Hz, 2 H, NCH_2_), 3.55 (q, ^3^*J*_H,H_ = 5.2 Hz, 2 H, *CH_2_*OH), 2.78 (t, ^3^*J*_H,H_ = 6.9 Hz, 1 H, *CH*(CH_3_)_2_), 2.69 (t, ^3^*J*_H,H_ = 6.6 Hz, 2 H, CH_2_CO), 1.03 (d, ^3^*J*_H,H_ = 6.9 Hz, 6 H, *CH(CH_3_)_2_*) ppm. ^13^C NMR (75 MHz, [D_6_]DMSO): *δ* = 175.65, 170.76, 160.29, 149.14, 133.93, 113.93, 66.02, 58.84, 44.48, 33.79, 32.81, 19.52 (2 C) ppm. C_13_H_19_N_3_O_6_ (313.31): calcd. C 49.84, H 6.11, N 13.41; found C 50.15, H 6.06, N 13.09.

**3-(3,4-Dihydro-5-methyl-2,4-dioxopyrimidin-1(2*H*)-yl)propanoic acid (3):**

Michael-type adduct of uracil to HEA (**2a**) (1 mmol) was refluxed in 5% HCl_aq_ (8 ml). During first 5 minutes of refluxing solid phase disappeared. During the next 15 minutes of boiling neo-crystalline material appeared. The mixture was then cooled to room temperature, which resulted in further precipitation of fine-formed crystals. The post-reaction mixture was kept for 16 h in refrigerator. Afterwards crystals were filtered off and rinsed with ice-water to neutral pH.

Colourless needles; yield 82%; m.p. 172-174 ºC, lit. m.p. 174-175 ºC;[17] ^1^H NMR (300MHz, [D_6_]DMSO): *δ* = 12.41 (s, 1 H, COOH), 11.23 (s, 1 H, N3-H), 7.49 (d, 1 H, H6, ^4^*J*_H-H_ = 0.9 Hz), 3.81 (t, 2 H, >NC*H*_2_, ^3^*J*_H-H_ = 6.9 Hz), 2.59 (t, 2 H, C*H*_2_, ^3^*J*_H-H_ = 6.9 Hz), 1.73 (s, 3H, Me) ppm. ^13^C NMR: δ= 12.0; 32.9; 44.0; 108.2; 141.9; 150.8; 164.4; 172.4 ppm.

**3-(5-Methyl-2,4-dioxo-3,4-dihydro-2*H*-pyrimidin-1-yl)-propionic acid 2-[3-(5-bromo-2,4-dioxo-3,4-dihydro-2*H*-pyrimidin-1-yl)-propionyloxy]-ethyl ester (4) (Figure 1):**

To a suspension of **3** (1 mmol) in THF (8 ml) was added dropwise *N*-methylmorpholine (1.1 mmol), and after 10 minutes of stirring at room temperature **2d** (1.1 mmol) and DMT-MM (1.1 mmol) were added. The progress of reaction was monitored by TLC (10% vol. MeOH/CHCl_3_). After 48 h the solvent was removed under reduced pressure and the residue was purified on column chromatography (10% vol. MeOH/CHCl_3_).

Figure 1. Protons assignment in NMR spectrum of the model ester-conjugated nucleoside (**4**)

Colourless needles; yield 88%; m.p. 137-138 ºC; ^1^H NMR (300MHz, CDCl_3_): *δ* = 10.14 (s, 1 H, H3’), 9.73 (s, 1 H, H3), 7.80 (s, 1 H, H6’), 7.19 (d, 1 H, H6, ^4^*J*_H-H_ = 0.9 Hz), 4.30 (s, 4 H, H10’ and H11’), 4.02 (t, 2 H, H7’, ^3^*J*_H-H_ = 5.7 Hz), 3.97 (t, 2 H, H7, ^3^*J*_H-H_ = 6.3 Hz), 2.81 (q, 4 H, H8’ and H8, ^3^*J*_H-H_ = 6.3 Hz, ^3^*J*_H-H_ = 5.7 Hz), 1.91 (d, 3 H, CH_3_, ^4^*J*_H-H_ = 0.9 Hz) ppm. ^13^C NMR: *δ* = 12.4, 32.8, 33.0, 45.1, 45.7, 62.5, 62.8, 96.0, 96.2, 110.5, 141.7, 145.4, 150.4, 151.1, 164.9, 171.2, 171.4. C_17_H_19_BrN_4_O_8_ (487.26): calcd. C 41.90, H 3.93, N 11.50; found C 42.01, H 3.86, N 11.38.
